# Adaptable test bench for ASTM-compliant permeability measurement of porous scaffolds for tissue engineering

**DOI:** 10.1038/s41598-024-52159-4

**Published:** 2024-01-19

**Authors:** Stefano Gabetti, Beatrice Masante, Alessandro Schiavi, Elisa Scatena, Eleonora Zenobi, Simone Israel, Alessandro Sanginario, Costantino Del Gaudio, Alberto Audenino, Umberto Morbiducci, Diana Massai

**Affiliations:** 1 Department of Mechanical and Aerospace Engineering and PolitoBIOMed Lab, Politecnico di Torino, Corso Duca Degli Abruzzi, 24, 10129 Turin, Italy; 2 Centro 3R, Interuniversity Center for the Promotion of the 3Rs Principles in Teaching and Research, Turin, Italy; 3 Department of Surgical Sciences, CIR-Dental School, University of Turin, Turin, Italy; 4 Applied Metrology and Engineering Division, INRiM-National Institute of Metrological Research, Turin, Italy; 5 E. Amaldi Foundation, Rome, Italy; 6 Department of Electronics and Telecommunications, Politecnico di Torino, Turin, Italy; 7 Italian Space Agency, Rome, Italy

**Keywords:** Engineering, Biomedical engineering

## Abstract

Intrinsic permeability describes the ability of a porous medium to be penetrated by a fluid. Considering porous scaffolds for tissue engineering (TE) applications, this macroscopic variable can strongly influence the transport of oxygen and nutrients, the cell seeding process, and the transmission of fluid forces to the cells, playing a crucial role in determining scaffold efficacy. Thus, accurately measuring the permeability of porous scaffolds could represent an essential step in their optimization process. In literature, several methods have been proposed to characterize scaffold permeability. Most of the currently adopted approaches to assess permeability limit their applicability to specific scaffold structures, hampering protocols standardization, and ultimately leading to incomparable results among different laboratories. The content of novelty of this study is in the proposal of an adaptable test bench and in defining a specific testing protocol, compliant with the ASTM International F2952-22 guidelines, for reliable and repeatable measurements of the intrinsic permeability of TE porous scaffolds. The developed permeability test bench (PTB) exploits the pump-based method, and it is composed of a modular permeability chamber integrated within a closed-loop hydraulic circuit, which includes a peristaltic pump and pressure sensors, recirculating demineralized water. A specific testing protocol was defined for characterizing the pressure drop associated with the scaffold under test, while minimizing the effects of uncertainty sources. To assess the operational capabilities and performance of the proposed test bench, permeability measurements were conducted on PLA scaffolds with regular (PS) and random (RS) micro-architecture and on commercial bovine bone matrix-derived scaffolds (CS) for bone TE. To validate the proposed approach, the scaffolds were as well characterized using an alternative test bench (ATB) based on acoustic measurements, implementing a blind randomized testing procedure. The consistency of the permeability values measured using both the test benches demonstrated the reliability of the proposed approach. A further validation of the PTB’s measurement reliability was provided by the agreement between the measured permeability values of the PS scaffolds and the theory-based predicted permeability value. Once validated the proposed PTB, the performed measurements allowed the investigation of the scaffolds’ transport properties. Samples with the same structure (guaranteed by the fused-deposition modeling technique) were characterized by similar permeability values, and CS and RS scaffolds showed permeability values in agreement with the values reported in the literature for bovine trabecular bone. In conclusion, the developed PTB and the proposed testing protocol allow the characterization of the intrinsic permeability of porous scaffolds of different types and dimensions under controlled flow regimes, representing a powerful tool in view of providing a reliable and repeatable framework for characterizing and optimizing scaffolds for TE applications.

## Introduction

Tissue engineering (TE) approaches aim at developing in vitro functional substitutes of native tissues^1^. The effective development of engineered tissues requires the substantial recapitulation of the interactions between cells and their microenvironment, which is characterized by a tissue-specific three-dimensional (3D) architecture^2^. In particular, the structure of the biological tissues is composed of two main regions: the vascular space, consisting of blood and lymphatic vessels; and the extravascular area, which is a porous medium, including the cells and the extracellular matrix (ECM), saturated by interstitial fluid^3^. In the extravascular space, cells reside in the ECM pores, which can be interlinked and form channels for the transport of nutrients, metabolites, inhibitors, and other signaling molecules. Therefore, TE strategies are often based on the use of porous substrates, called scaffolds, designed to provide a biomimetic 3D architecture with the aim to guarantee mechanical support and to promote cell colonization, migration, and proliferation, while also ensuring adequate oxygen and nutrient uptake and the removal of metabolic wastes^4^. The structural characteristics of the scaffolds, such as porosity, pore size and distribution, tortuosity, and specific surface area, concurrently influence the transport phenomena, with effects on cell attachment, cell migration, and tissue in-growth^5–8^. However, the characterization of TE porous scaffolds in terms of single microscopical quantities is not fully indicative of the scaffold biomimetic functional properties, as they cannot provide a clear correlation with transport phenomena and cell behavior, if taken individually^9,10^. Moreover, the in-depth analysis of the scaffold microstructure requires cumbersome and expensive techniques, such as electron microscopy and micro-computed tomography (micro-CT) and needs advanced and time-consuming image processing techniques for measuring the actual path length of porous microchannels^11,12^.

Differently, the intrinsic permeability, which is a macroscopic material property describing the ability of a porous medium to be penetrated by a fluid, reflects the role of the microscopic structure parameters mentioned above and can be measured with conventional equipment^13–15^. As an integral parameter, intrinsic permeability can be used as a quantitative descriptor related to scaffold biomimetic properties^11^. Indeed, it directly affects pressure and shear forces inside the scaffolds^16^, which are fundamental stimuli determining effective cell seeding, cellular differentiation, tissue formation, and scaffold degradation rate^17–22^. Moreover, in view of a consistent manufacturing process, permeability measurements can support the optimization of the structure of TE porous scaffolds and can be exploited for quality assurance purposes. For these reasons, intrinsic permeability has been widely used for characterizing TE porous scaffolds and several systems for direct evaluation of scaffolds permeability have been developed during the past decades, adopting different test fluids^23^. For example, test benches exploiting the airflow through the scaffold allow quick measurements and were adopted to characterize the intrinsic permeability in dry conditions^11,22,24,25^. However, TE scaffolds are designed to work under physiologically relevant hydrated conditions and often respond with swelling, which can influence their structure, shape, and mechanical properties depending on the materials used for their fabrication^26,27^. Therefore, an experimental assessment performed under wet conditions could provide a more lifelike characterization of the behavior of the scaffolds once implanted. Test benches based on a liquid as a test fluid were developed adopting two approaches: gravity-based and pump-based methods^23^. The gravity-based method is centered on the application of a known pressure head of liquid and on the measurement of the flow rate through the tested sample^9,28^. It is commonly used for characterizing scaffolds with high permeability values^29,30^ (10^–12^–10^–8^ m^2^), while it proved to be unsuitable for low permeable samples, as pressure heads of several meters would be required to induce a detectable flow rate. The pump-based method, relying on the measurement of a pressure drop across the sample under an imposed flow rate, allows the investigation over a wide range of permeability values^19,31,32^ (10^–15^–10^–9^ m^2^), also enabling to impose different specific flow regimes^33^.

Despite the numerous test benches and methods described in the literature, a measurement standard is still missing and the absence of standardized testing procedures make unfeasible the comparison of the results achieved in different laboratories^23^. To address this issue, in 2022, the American Society for Testing and Materials International (ASTM International) published the F2952 standard, which prescribes the guidelines for determining the mean Darcy permeability coefficient for a porous scaffold for TE applications^34^. Based on the previous literature, the guidelines highlight the importance of optimizing the design of the test bench and of the testing procedures for obtaining reliable and comparable measurements, unaffected by experimental artifacts.

Inspired by the ASTM International F2952 standard, we developed a closed-loop permeability test bench exploiting the pump-based method and we devised a specific testing protocol for measuring the permeability of TE porous scaffolds. Following the proposed approach, the experimental permeability values were calculated with their relative uncertainty and were provided in a defined range within a confidence level of 95%. For assessing the operational capabilities and performance of the developed test bench, the permeability of three different types of scaffolds for bone TE, i.e., 3D-printed scaffolds with regular geometry, 3D-printed scaffolds with random microarchitecture, and commercial bovine-derived scaffolds, was measured. To validate the proposed approach, a comparison study was performed assessing the compatibility of the whole set of measured permeability values with those obtained on the same scaffolds using a previously developed test bench based on acoustic pressure recordings^35,36^. As regards the reliability of the testing protocol, the permeability measurements of the scaffolds with regular geometry were compared with the results of the Kozeny-Carman theory-based approach, taking into consideration the scaffold geometrical features. Finally, to assess the suitability of the tested scaffolds for bone TE applications, their permeability values were compared with permeability values of native bone.

## Materials and methods

### Permeability test bench

The permeability test bench (PTB) was developed taking into account the basic principles to be followed to assess the mean Darcy permeability coefficient reported in the ASTM F2952-22 guidelines, which are: (i) to ensure complete wetting of the sample, avoiding the persistence of trapped air bubbles in the structure; (ii) to clamp the sample and guarantee watertightness, while avoiding excessive deformation; (iii) to maintain a constant pressure head during the test; (iv) to select a pressure measurement apparatus with high sensitivity; (v) to perform multiple pressure and flow rate readings alternated by time lags to allow the system to reach a steady-state condition^34^. Moreover, the PTB was designed to fulfil further specific requirements: to be versatile for testing samples of different sizes under physiologically relevant hydrated conditions; to guarantee the development of a well-known flow regime inside the tested sample; to allow the measurement of a wide range of permeability values, considering that permeability of biological materials and scaffolds can range from 10^–22^ to 10^–8^ m^2^^37–41^; to enable long-term tests for analyzing the impact of scaffold’s degradation over time. Considering all these requirements (Table 1), a pump-based PTB was designed. In detail, the PTB is composed of a permeability chamber for housing the sample, a hydraulic circuit for imposing a controlled flow rate through the sample, and a pressure measurement unit for measuring the pressure drop due to the sample.

**Table 1:** Requirements considered for the PTB design and the measurement procedure.

	Requirements	Solutions
Basic principles from ASTM F2952-22 guidelines	Complete sample wetting and air bubble removal	Hydraulic circuit with vertical set-up and upwards flow direction to promote the air bubble removal; transparent tubing for visual checking
	Watertight sample clamping	Press-fit tailored gasket and additional wrapping around the sample with Teflon tape
	Constant pressure head	Hydraulic circuit with vertical set-up with free-surface reservoir located at the highest position with respect to the permeability chamber
	High sensitivity pressure measurement apparatus	High sensitivity pressure sensors (5 µV/V/mmHg) and high resolution DAQ (24 bit)
	Multiple readings under steady-state condition	Hydraulic circuit with closed-loop set-up to perform continuous measurements; flow rate control with the peristaltic pump to impose constant flow rate
Specific PTB requirements	Testing samples of different sizes under hydrated conditions	Tailored flexible gaskets manufactured by using customized modular moulds to house samples of different geometry and size; hydraulic circuit with closed-loop set-up for recirculating the testing fluid and guaranteeing hydrated conditions
	Controlled flow regime inside the tested sample	Flow rate control with the peristaltic pump to impose defined flow rate and permeability measurement procedure including an a priori evaluation of the interstitial *Re*
	Measure of permeability over a wide range of values	Peristaltic pump with wide range of flow rate (0.8–480.0 mL/min) and pressure sensors characterized by a wide measurement range (−30 to 300 mmHg)
	Possibility to perform long-term tests	Hydraulic circuit with closed-loop set-up

#### Permeability chamber

**Figure 1:**
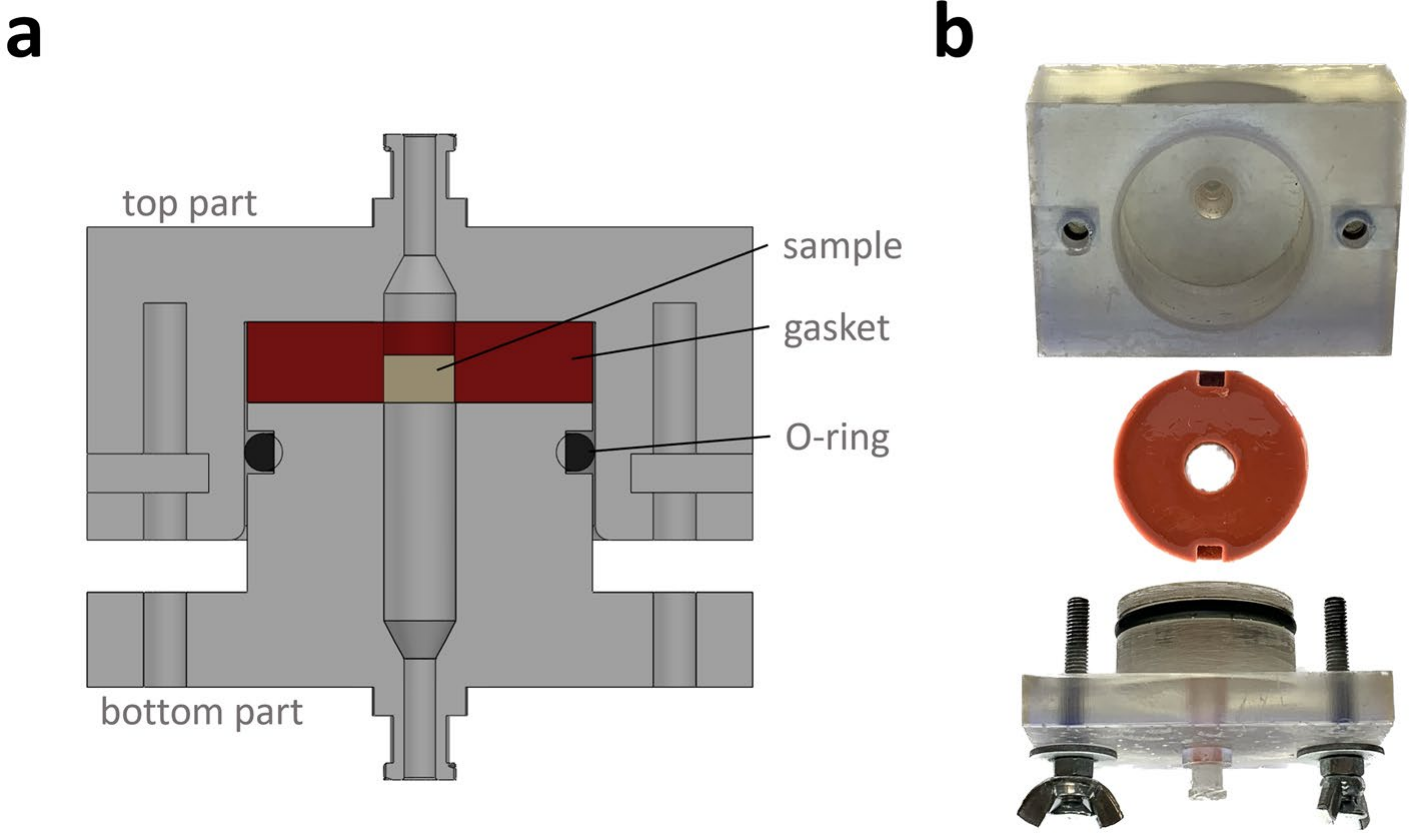
**(a)** Section view of the assembled PC. **(b)** Picture of the PC components: 3D-printed top and bottom parts, and silicone gasket.

The main component of the PTB is the permeability chamber (PC), designed for housing samples of different geometries and sizes. In detail, the PC, drawn by using Solidworks (Dassault Systemes, France) and manufactured by stereolithography (Clear Resin, 3D printer Form 3, FormLabs, USA), has a parallelepiped shape (length = 70.0 mm, width = 50.0 mm, height = 62.6 mm) and consists of two parts, coupled by screws, with an internal cylindrical geometry (Fig. 1). The top part is designed for housing samples (height = 1–14 mm, diameter or side = 8–27 mm) to be press-fit into tailored interchangeable flexible gaskets. An O-ring placed circumferentially in the bottom part guarantees watertightness (Fig. 1a). The PC has a central channel (diameter = 8 mm), which allows the fluid flowing through the sample, and integrated luer lock connections enabling its plugging into the hydraulic circuit (Fig. 1b). For the flexible gasket manufacturing, a dedicated modular mould with interchangeable spacers was fabricated in acrylonitrile–butadiene–styrene (ABS) by fused deposition modelling (3D printer uPrint SE, Stratasys, USA). Depending on the sample geometry and size, flexible gaskets with central channels of different shapes and sizes can be produced by inserting different interchangeable spacers in the mould prior to casting the liquid silicone rubber (R Pro Tech 33, Reschimica, Italy). Additional details on the modular mould assembly and gasket manufacturing procedure are provided in the Supplementary Materials. For this study, in which cylindrical scaffold samples were tested, 4 flexible gaskets were manufactured inserting in the mould 4 different cylindrical spacers (external diameter = 9.5–10 mm) and then pouring the silicone into the mould.

#### Hydraulic circuit and pressure measurement unit

**Figure 2:**
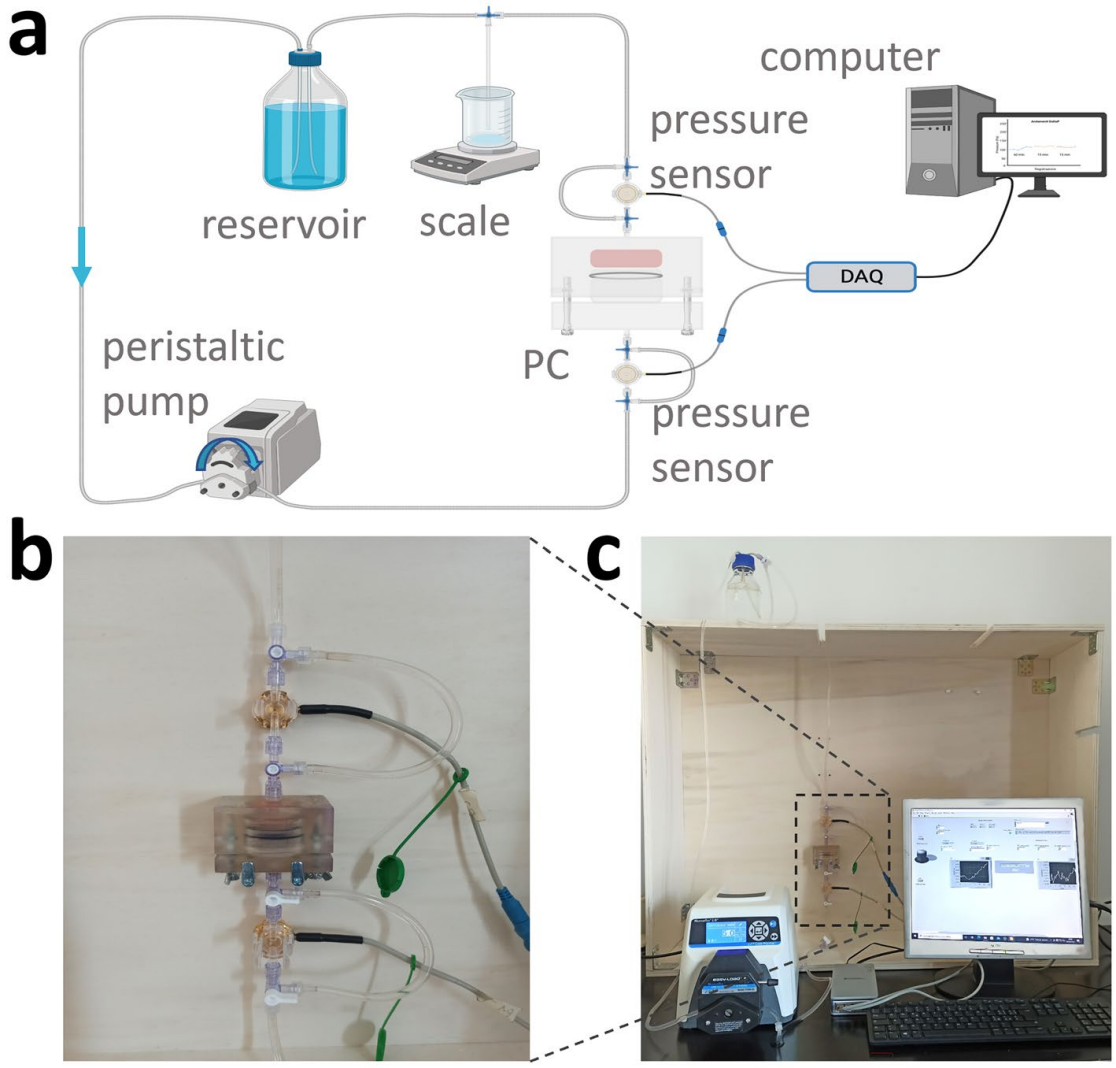
**(a)** Schematic drawing of the PTB with its components (created with BioRender). **(b)** Picture of the PC and the pressure sensors in the PTB set-up, showing the cables for signal acquisition and the tubing for air bubble removal. **(c)** Picture of the PTB set-up.

The PC is connected to a closed-loop hydraulic circuit, aimed at imposing a controlled flow rate through the sample. The circuit is composed of a reservoir, a peristaltic pump (Masterflex, Cole-Parmer, USA), impermeable transparent tubing (internal diameter = 3.2 mm; Tygon S3, Saint-Gobain, France), three-way stopcocks, and it also includes two in-line physiological relative pressure sensors (SP844, HJK Sensoren, Germany) connected to the pressure measurement unit (Fig. 2a). The selected pump and tubing enable to impose flow rates in the range of 0.8–480.0 mL/min. As test fluid, demineralized water flowing from bottom to top is used. The pressure sensors, characterized by a measurement range between −30 and 300 mmHg and a sensitivity of 5 µV/V/mmHg, are located upstream and downstream the PC for measuring the pressure drops across it. Additional tubing allows the removal of possible air bubbles trapped inside the sensor membranes (Fig. 2b). The PC, the pressure sensors, and the reservoir are mounted in-line and vertically on a support structure, with the free-surface reservoir located at the highest position with respect to the PC, to facilitate the removal of possible air bubbles and to impose a constant pressure head (Fig. 2c). Sensor output signals are acquired by a 24 bit data acquisition (DAQ) system (NI9237 module connected to a cDAQ9191, National Instruments, USA), which is controlled by a computer running a purpose-built software with a LabView interface for recording the measured pressure data and a Matlab (Mathworks, USA) script for post-processing the recorded data. A balance scale (PS1000.R2, Radwag, Poland) characterized by a measurement range of 1 kg and a resolution of 1 mg is used to measure the flow rate at the end of the test.

### Permeability measurements and comparisons

**Figure 3:**
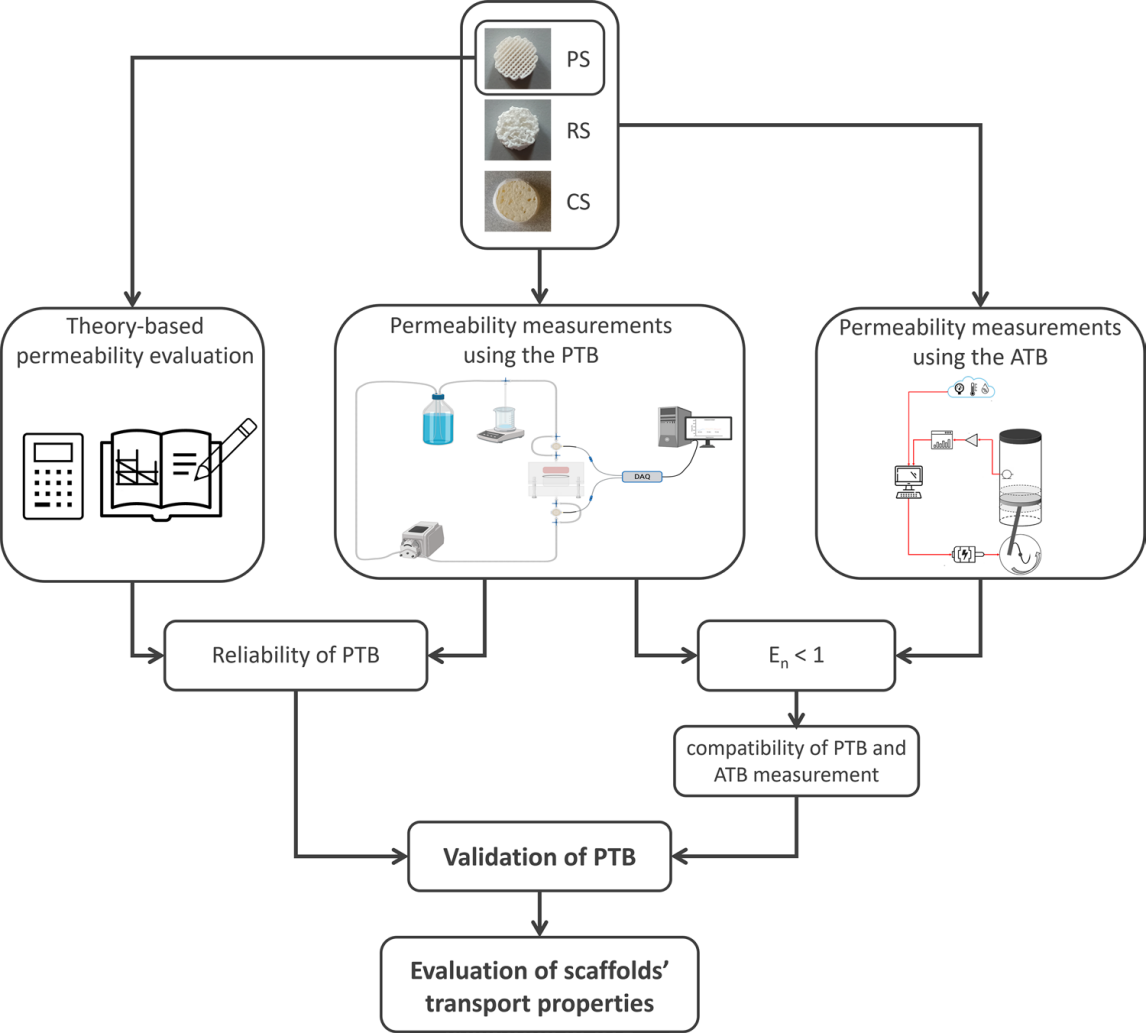
Workflow of the performed permeability measurements and comparisons.

The operational capabilities and performance of the developed PTB were assessed by testing different types of scaffolds and comparing the measured permeability values with those obtained using both an alternative permeability test bench (ATB) based on an acoustic method^35^ and a theory-based approach focused on transport in porous media (for the most regular scaffold type solely). Figure 3 summarizes the workflow of the performed measurements and comparisons. In the following, details about the tested scaffolds, the permeability measurement procedures, the data analysis, the comparison criteria, and the theory-based evaluation are provided.

#### Scaffold models

**Figure 4:**
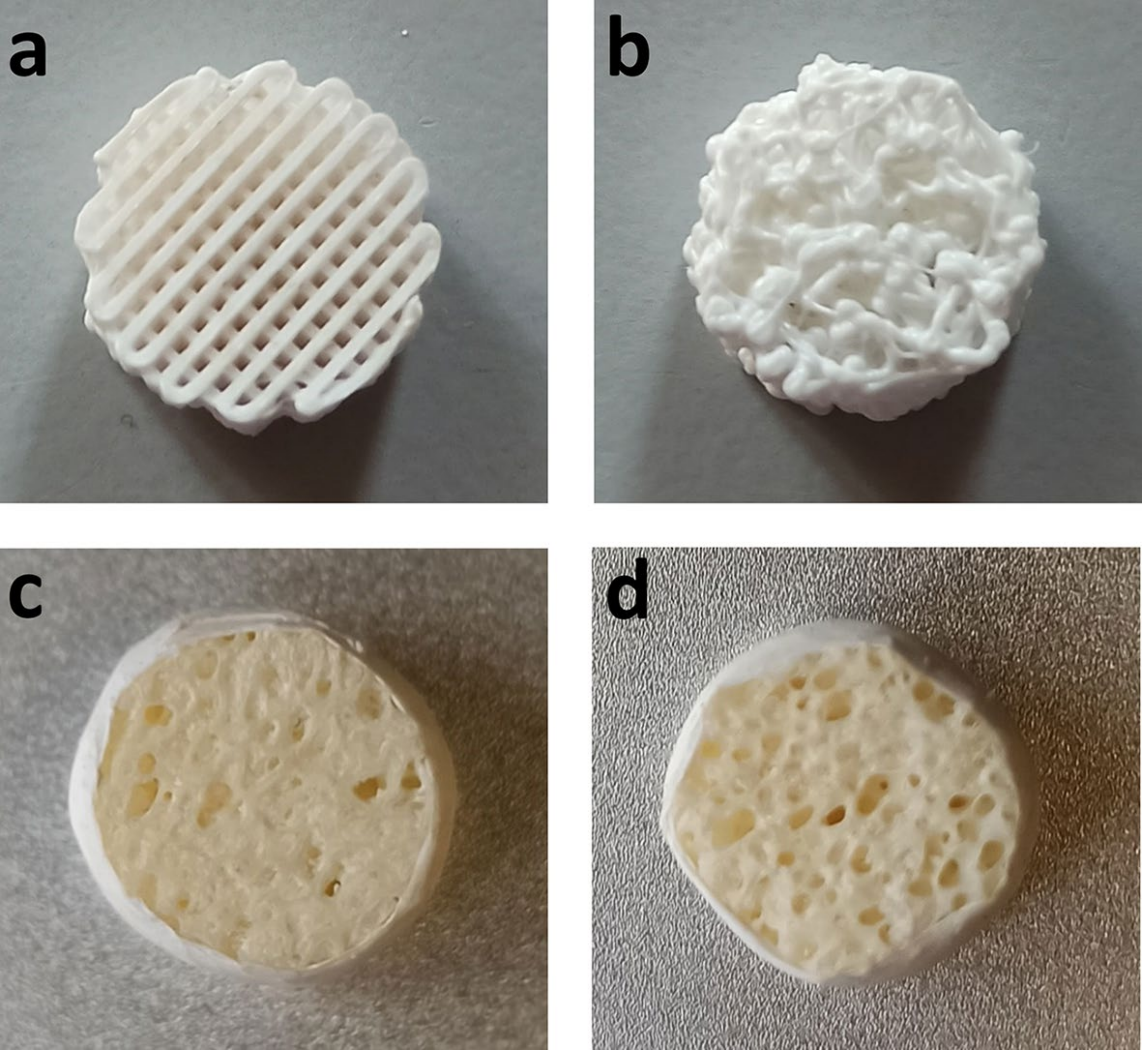
Bone TE scaffolds tested in the study: **(a)** Explanatory 3D-printed perpendicular scaffold (PS). **(b)** Explanatory 3D-printed random scaffold (RS). **(c)** Commercial scaffold sample 1 (CS1). **(d)** Commercial scaffold sample 2 (CS2).

For testing the PTB, 3D-printed poly-lactic acid (PLA) scaffolds and commercial ones were selected. In detail, PLA scaffolds were fabricated by fused deposition modelling (Raise3D N2 3D Printer, Raise 3D Inc., USA), using a PLA filament (FILOALFA, Italy) extruded through a nozzle (diameter = 0.4 mm) at 205 °C and delivered on the build platform at 60 °C. For the study, two different structures were proposed: (i) an ordered lattice of cross-plied fibers intersecting perpendicularly (Fig. 4a), referred to as ‘perpendicular scaffold’ (PS), and (ii) a random microarchitecture designed to mimic the trabecular bone structure^42,43^ (Fig. 4b), referred to as ‘random scaffold’ (RS). The PS and RS scaffolds are the result of two specific and unique G-code files that drive the 3D printer, aimed to deal with constructs prepared adopting the same processing conditions. The G-code files for the PS and RS scaffolds were generated at the end of the design process and then repeatedly used to fabricate scaffolds characterized by analogue geometrical features, respectively. For each model, two cylindrical PLA scaffolds were manufactured (design dimensions: diameter = 10 mm; thickness = 5 mm; nominal pore size = 400 μm and ordered lattice for PS; nominal porogen size = 600 μm and random lattice for RS) and labelled PS1, PS2, RS1, and RS2, respectively. An optical microscopy (Nikon Eclipse 80i) session was carried out to experimentally evaluate the mean pore diameter (*D*) of the PS model, being *D*_*PS*_ = 401.4 ± 16.9 μm.

Furthermore, commercial scaffolds for bone TE (SmartBone IBI S.A., Switzerland), based on bovine-derived mineral matrices combined with bioresorbable polymers and collagen fragments, were selected. In particular, two scaffolds were cut in a cylindrical shape and labelled CS1 (Fig. 4c) and CS2 (Fig. 4d), respectively, with a mean pore diameter* D*_*CS*_ = 378 ± 145 μm ^44^.

#### Permeability measurement procedure using the PTB

A specific protocol was defined for the permeability measurement with the proposed PTB. Firstly, each scaffold sample was measured with a caliper to take into account its actual dimensions. The sample was then inserted press-fit within a tailored flexible gasket and, in case of clearance between the sample and the gasket, it was further wrapped with Teflon tape to prevent water leakage. The sample-gasket assembly was then inserted into the PC top part, the bottom part was coupled, the PC was closed by tightening the screws, and finally it was connected to the hydraulic circuit. To fill the hydraulic circuit, 500 mL of demineralized water at room temperature were poured in the reservoir and the pump was activated, visually checking the filling through the transparent tubing.

As regards the pump flow rate for the permeability measurement test, it was defined considering that for applying the Darcy law a laminar flow regimen should be guaranteed within each sample. This is ensured when the interstitial Reynolds number (*Re*) satisfies the condition^24,45–47^:

1 $$Re = \frac{\rho v D}{\mu }<1$$

where *v* is the fluid linear velocity, *D* is the mean pore diameter of the sample, *ρ* is the fluid density (*ρ* = 998 kg/m^3^), and *μ* is the fluid dynamic viscosity (*μ* = 1.002 mPa·s). Thus, taking into account the mean pore diameter values and the measured geometrical features of the selected scaffolds (reported in Table 2), a flow rate of 5 mL/min was identified, corresponding for the selected scaffolds to Reynolds numbers, respectively: *Re*_*PS*_ = 0.424 ± 0.018; *Re*_*RS*_ = 0.634; *Re*_*CS*_ = 0.399 ± 0.153. Moreover, to ensure that the sample was completely wet before the test and the air removed, the pump was run overnight. Before starting each test, the pump was stopped and the pressure sensors, located respectively upstream and downstream to the PC, were zeroed to neglect pressure differences due to their different heights. For each scaffold, a total of four permeability measurement tests were performed repeating the same procedure, and during each test five pressure measurements were recorded, initially over a period of 1 h and subsequently over four periods of 15 min (Supplementary Fig. S1). For each measurement, the total pressure drop across the PC (*Δp*_*total*_) was calculated as the difference between the average pressure values recorded by the two sensors. Moreover, for obtaining the pressure drop solely due to the PC geometry (*Δp*_*PC*_), five measurements were performed following the same procedure but without the sample inserted. Consequently, the pressure drop due to the sample (*Δp*_*sample*_) was calculated as:

**Table 2:** Measured and calculated parameters for all selected scaffolds by using the proposed experimental and theory-based approaches.

		Parameter	PS1	PS2	RS1	RS2	CS1	CS2
Measurement	Sample	*L* (m)	(4.91 ± 0.04) × 10^–3^	(4.95 ± 0.05) × 10^–3^	(5.13 ± 0.01) × 10^–3^	(5.26 ± 0.13) × 10^–3^	(3.58 ± 0.05) × 10^–3^	(4.66 ± 0.06) × 10^–3^
		*A* (m^2^)	(7.82 ± 0.18) × 10^–5^	(8.03 ± 0.01) × 10^–5^	(7.60 ± 0.24) × 10^–5^	(7.51 ± 0.34) × 10^–5^	(8.17 ± 0.54) × 10^–5^	(7.90 ± 0.39) × 10^–5^
	PTB	*Q* (ml/min)	4.84 ± 0.31	4.67 ± 0.16	4.69 ± 0.08	4.58 ± 0.19	4.90 ± 0.02	4.99 ± 0.02
		*Δp*_*sample*_ (Pa)	14.9 ± 2.58	10.8 ± 3.35	22.1 ± 4.32	22.9 ± 5.13	124.0 ± 12.4	63.5 ± 16.1
Calculation	Theory-based approach	***k*** (m^2^)	3.95 × 10^–10^					
	PTB	***k* ± *U***_***k***_ (m^2^)	(3.39 ± 0.63) × 10^–10^	(4.44 ± 1.38) × 10^–10^	(2.39 ± 0.47) × 10^–10^	(2.33 ± 0.54) × 10^–10^	(2.89 ± 0.35) × 10^–11^	(7.73 ± 2.00) × 10^–11^
	ATB	***k* ± *U***_***k***_ (m^2^)	(3.70 ± 0.31) × 10^–10^	(4.35 ± 0.45) × 10^–10^	(2.07 ± 0.27) × 10^–10^	(2.33 ± 0.29) × 10^–10^	(2.63 ± 0.23) × 10^–11^	(8.81 ± 0.93) × 10^–11^
	PTB vs ATB	*E*_*n*_	0.45	0.06	0.58	0	0.62	0.49

2 $${\Delta p}_{sample}= {\Delta p}_{total} {- \Delta p}_{PC}$$

At the end of each test, to guarantee permeability measurement accuracy, the flow rate was measured by opening the circuit downstream of the PC and measuring the volume of water flowing in a defined time interval (2 min) by using the scale.

Finally, the intrinsic permeability (*k*) of each sample was obtained from the Darcy law:

3 $$k=\mu \frac{Q}{{\Delta p}_{sample}}\frac{L}{A}$$

where *Q* is the flow rate, *L* is the thickness of the sample, and *A* is the cross-sectional area of the sample. For each tested sample, the mean and the standard deviation of each parameter experimentally measured, i.e., *L*, *A*, *Δp*_*sample*_, and *Q*, were calculated. The mean permeability coefficient *k* of each sample was then calculated applying Darcy equation (Eq. 3), considering the mean value of each parameter.

#### Permeability measurement procedure using the ATB

To assess the proposed PTB's reliability, an alternative permeability test bench (ATB), previously developed^35^, was adopted for testing all the samples. Briefly, the ATB is based on an acoustic method and consists of a closed cavity with a sample holder, a piston, and a low-frequency pressure field microphone that allows performing permeability measurements in dry conditions. As a first step, the test cavity was closed with an airtight lid and the microphone was calibrated performing pressure measurements of the sinusoidal pressure oscillations caused by the oscillating piston. Subsequently, for each measurement the sample was placed in the holder, to act as one of the walls of the cavity, and a sinusoidal volumetric airflow was generated in the cavity. The microphone measured the sinusoidal pressure component in the closed volume in which the air was subjected to a slow cycle of compression and rarefaction. The pressure wave drop was determined by the ratio ζ between the amplitude of the pressure wave measured in the hermetically closed air volume and the amplitude of the pressure wave measured in the same volume of air enclosed by the sample.

The permeability was determined by the relation between the root mean square volumetric airflow rate *q*_*v,rms*_ and the root mean square dynamic pressure measured in the closed cavity *p*_*rms*_, according to the Darcy’s law for oscillating flows with laminar regime^35^:

4 $$k=\mu \frac{Q}{\Delta P}\frac{L}{A}= \mu \frac{{q}_{v,{\text{rms}}}}{{p}_{{\text{rms}}}}\frac{L}{A}\zeta = \mu \frac{\omega {V}_{0}}{\gamma {p}_{0}}\frac{L}{A}\zeta$$

where *ω* is the airflow pulsation, *V*_*0*_ is the test cavity volume, *p*_*0*_ is the atmospheric pressure, *γ* is the air heat capacity ratio (*γ* = 1.4).

For each tested sample, the mean and the standard deviation of each parameter experimentally measured, i.e., *L*, *A*, *ω*, *V*_*0*_, *p*_*0*_ and ζ, were calculated. The mean permeability coefficient *k* of each sample was then calculated applying Eq. (4), considering the mean value of each parameter. Additional details on the ATB and testing procedure are provided in the Supplementary Materials.

#### Data analysis

To obtain a range of permeability values within a confidence level of 95%, the extended uncertainty was calculated in accordance with the guidelines developed by the International Bureau of Weights and Measures (BIPM)^48^. Technically, the budget of uncertainty was calculated according to the uncertainty propagation formula, considering the five contributions of uncertainties associated with fluid viscosity, flow rate, pressure drop due to the sample, and thickness and cross-sectional area of the sample, as follows:

5 $${s}_{k}=\sqrt{{\left(\frac{\partial k}{\partial \mu }\right)}^{2}\cdot {s}_{\mu }^{2}+{\left(\frac{\partial k}{\partial Q}\right)}^{2}\cdot {s}_{Q}^{2}+{\left(\frac{\partial k}{\partial \Delta p}\right)}^{2}\cdot {s}_{\Delta p}^{2}+{\left(\frac{\partial k}{\partial L}\right)}^{2}\cdot {s}_{L}^{2}+{\left(\frac{\partial k}{\partial A}\right)}^{2}\cdot {s}_{A}^{2}}$$

where *∂k/∂x* is the partial derivative of *k* with respect to the parameter *x*, and *s*_*x*_ is the uncertainty on the value of the parameter, which for the measured parameters is their standard deviation (*σ*_*x*_).

For both the PTB and the ATB, Eq. (5) was expanded considering the permeability Eqs. (3) and (4), obtaining the following relations.

6 $$\begin{array}{cc}PTB:& {s}_{k}=\sqrt{{\left(\frac{Q}{{\Delta p}_{sample}}\frac{L}{A}\right)}^{2}\cdot {\sigma }_{\mu }^{2}+{\left(\frac{\mu }{{\Delta p}_{sample}}\frac{L}{A}\right)}^{2}\cdot {\sigma }_{Q}^{2}+{\left(-\mu \frac{Q}{{{\Delta p}_{sample}}^{2}}\frac{L}{A}\right)}^{2}\cdot {\sigma }_{{\Delta p}_{sample}}^{2}+{\left(\frac{\mu }{{\Delta p}_{sample}}\frac{Q}{A}\right)}^{2}\cdot {\sigma }_{L}^{2}+{\left(-\mu \frac{Q}{{\Delta p}_{sample}}\frac{L}{{A}^{2}}\right)}^{2}\cdot {\sigma }_{A}^{2}}\\ ATB:& {s}_{k}=\sqrt{{\left(\frac{\omega {V}_{0}}{\gamma {p}_{0}}\frac{L}{A}\zeta \right)}^{2}\cdot {\sigma }_{\mu }^{2}+{\left(\mu \frac{{V}_{0}}{\gamma {p}_{0}}\frac{L}{A}\zeta \right)}^{2}\cdot {\sigma }_{\omega }^{2}+{\left(\mu \frac{\omega }{\gamma {p}_{0}}\frac{L}{A}\zeta \right)}^{2}\cdot {\sigma }_{{V}_{0}}^{2}+{\left(-\mu \frac{\omega {V}_{0}}{\gamma {{p}_{0}}^{2}}\frac{L}{A}\zeta \right)}^{2}\cdot {\sigma }_{{p}_{0}}^{2}+{\left(-\mu \frac{\omega {V}_{0}}{\gamma {p}_{0}}\frac{L}{{A}^{2}}\zeta \right)}^{2}\cdot {\sigma }_{A}^{2}+ {\left(\mu \frac{{\omega V}_{0}}{\gamma {p}_{0}}\frac{L}{A}\right)}^{2}\cdot {\sigma }_{\zeta }^{2}}\end{array}$$

The extended uncertainty (*U*_*k*_) was then quantified as follows:

7 $${U}_{k}=c\cdot {s}_{k}$$

where *c* is the Student-t coverage factor, which for a confidence value of 95% is equal to 2. Thus, for the experimental measurements, the permeability values were reported with their corresponding extended uncertainties as follows:

8 $${k\pm U}_{k}$$

Permeability values of samples characterized by the same structure were compared performing a Welch's *t*-test using GraphPad Prism 8 (Dotmatics, USA).

#### Comparison criteria

A specific protocol was defined for the comparison of the scaffold permeability values obtained using the PTB and the ATB, according to methods and procedures currently used in applied metrology^49^. In detail, to minimize operator-dependent bias, the permeability measurements were conducted in blind: (i) the order followed for testing all the samples was randomized; (ii) each operator involved in the experimental measurement used either the PTB or the ATB; (iii) no results were disclosed until all the samples were tested. For each sample, the assessment of the metrological compatibility of measurement results^50^ between the two experimental approaches was carried out by calculating the normalized error (*E*_*n*_):

9 $${E}_{n}=\frac{\left|{k}_{1}-{k}_{2}\right|}{\sqrt{{U}_{{k}_{1}}^{2}+{U}_{{k}_{2}}^{2}}}$$

where *k*_*1*_ and *k*_*2*_ are the mean permeability values and *U*_*k1*_ and *U*_*k2*_ are the extended uncertainties obtained using the PTB and the ATB, respectively.

Properly, the normalized error *E*_*n*_ provides a statistical evaluation between two independent experimental results, including uncertainties, to evaluate the compatibility (or the congruity) of compared values, obtained from different experimental methods, techniques or measuring systems. The evaluation of *E*_*n*_ is routinely carried out for proficiency tests and for interlaboratory comparisons: if *E*_*n*_ < 1, the compared values can be considered compatible, not otherwise.

#### Theory-based evaluation of the permeability

The PS scaffolds, characterized by a regular structure, can be considered isotropic porous media and their permeability was evaluated according to the theory of porous media developed by Kozeny and Carman^51,52^, as follows:

10 $$k=\frac{{\varphi }^{3}}{{c}_{K} {\tau }^{2}{ S}^{2}}$$

where *φ* is the porosity,* τ* is the tortuosity, *S* is the specific surface area, and *c*_*K*_ is the semi-empirical Kozeny constant of the considered porous medium. As regards the porosity of PS scaffolds, *φ* was measured equal to 68.6% by adopting the gravimetric method, as described in the Supplementary Materials. The tortuosity of the PS scaffolds was evaluated using the logarithmic model proposed elsewhere^53,54^:

11 $$\tau = 1-a\cdot ln\left(\varphi \right)$$

where *a* is a parameter depending on the internal structure of the porous medium, which was determined adopting the approach proposed by Comiti and Renaud^55^, as described in the Supplementary Materials. The specific surface area of the PS scaffolds, characterized by parallelepiped-shaped pores with squared cross-section, was calculated as according to the formula^56,57^:

12 $$S= \varphi \frac{P}{{A}_{p}}= \varphi \frac{4l}{{l}^{2}}= \varphi \frac{4}{l}$$

where *P* is the perimeter of the pore cross-section, *A*_*p*_ is the area of the pore cross-section, and *l* is the length of the pore cross-section side (0.4 mm). Finally, *c*_*K*_ in Eq. (10) was set equal to 11, considering that the internal geometry of the PS scaffolds is characterized by a cross-plied fiber lattice and that it is crossed by the fluid perpendicular to the fibers^58^.

## Results

### Permeability measurements and comparisons

**Figure 5:**
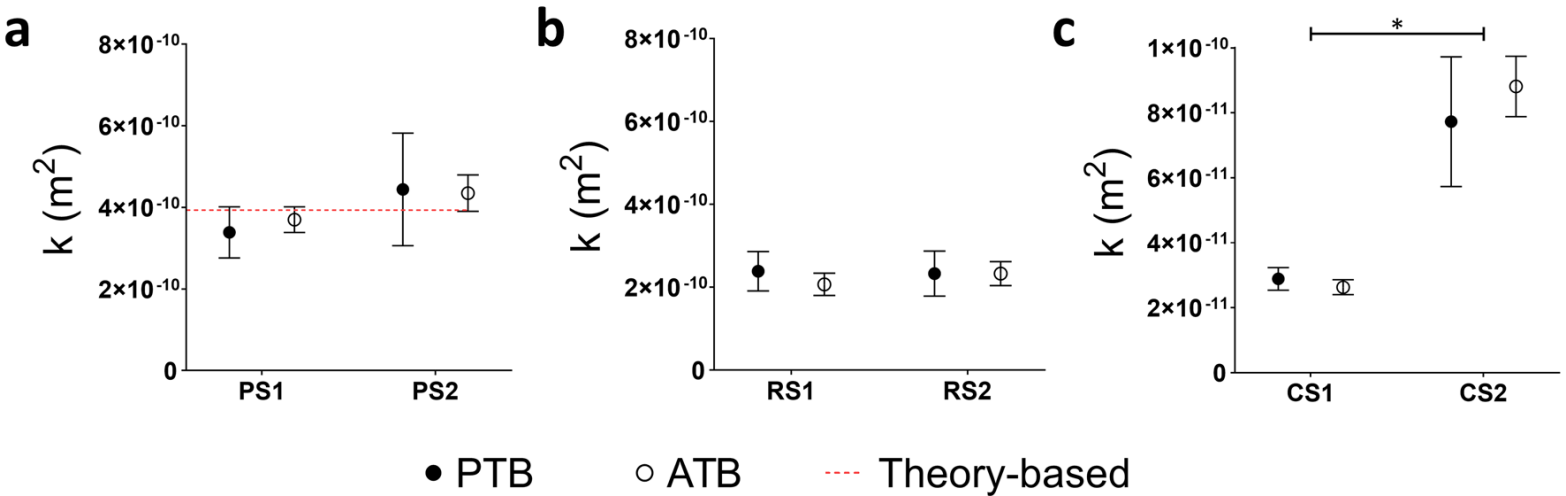
Permeability values obtained with the PTB and ATB for: **(a)** PS samples (red dotted line: theory-based permeability value). **(b)** RS samples. **(c)** CS samples. (*p* < 0.01 indicated by asterisk).

Permeability measurements and associated extended uncertainties obtained using the proposed PTB and the ATB are summarized in Table 2. For all the tested scaffolds (PS, RS, and CS), the permeability values were compatible between the two adopted test benches (Fig. 5), as confirmed by *E*_*n*_ values always lower than 1 (Table 2), demonstrating the reliability of the proposed PTB. This was further confirmed by the comparison of the theory-based permeability value (3.95 × 10^–10^ m^2^) and the experimental measurements obtained for the regular perpendicular scaffolds (PS1: (3.39 ± 0.63) × 10^–10^ m^2^; PS2: (4.44 ± 1.38) × 10^–10^ m^2^, Fig. 5a).

Once assessed the PTB reliability, the performed measurements allowed the investigation of the scaffolds’ transport properties (Fig. 3), revealing several features. Regarding the 3D-printed PLA scaffolds, the samples with the same structure were characterized by similar permeability values, as expected due to the high reproducibility of the specific manufacturing technique adopted (Fig. 5a, b). In detail, for the perpendicular scaffolds, the permeability values were (3.39 ± 0.63) × 10^–10^ m^2^ for PS1 and (4.44 ± 1.38) × 10^–10^ m^2^ for PS2. For the random scaffolds, permeability values were (2.39 ± 0.47) × 10^–10^ m^2^ for RS1 and (2.33 ± 0.54) × 10^–10^ m^2^ for RS2. Interestingly, the dimensional reproducibility of PS and RS scaffolds allowed to highlight the influence of the structure on the permeability: PS samples, exhibiting a regular geometry and straight pores, were characterized by higher permeability values compared to the RS samples (Fig. 4a, b). The commercial scaffolds, based on native bovine bone matrix and exhibiting heterogenous structure, were characterized by different permeability values (Fig. 5c): (2.89 ± 0.35) × 10^–11^ m^2^ for CS1 and (7.73 ± 2.00) × 10^–11^ m^2^ for CS2. The results confirmed what emerged by visual inspection (Fig. 4c, d), highlighting structural differences between the CS samples due to their biological origin.

Finally, considering the scaffold biomimicry, it was noted that the permeability values of CS and RS samples were in agreement with the values reported in the literature for bovine femoral cancellous^59,60^ (1.1 × 10^−11^–2.33 × 10^–10^ m^2^) and vertebral trabecular^61^ (1.63 ± 0.80 × 10^–10^ m^2^) bone and for human femoral trabecular^62^ bone (1.2 ± 1.1 × 10^–10^ m^2^).

## Discussion

Intrinsic permeability is a key determinant of the efficacy of porous scaffolds for TE applications. This property can indeed significantly influence the transport of oxygen and nutrients, the efficiency of the cell seeding process, and the transmission of appropriate physical stimuli (in particular shear stresses) to the embedded cells^22^. Therefore, the accurate measurement of the intrinsic permeability of porous scaffolds could represent an essential step in their optimization process.

Several methods have been proposed to characterize the scaffold permeability^9,11,19,24,25,28–33^, however, a standardized protocol is missing also because the different proposed measurement techniques limit their applicability to specific scaffold structures, resulting in not comparable results^23^. Regarding the testing conditions, it is important to consider that TE scaffolds, whether designed for laboratory (in vitro) use or clinical applications, are typically used under physiologically relevant hydrated conditions, which can influence their structure and consequently their permeability. Inspired by the need to provide a method for reliable and repeatable permeability measurements of TE scaffolds under usage-like conditions and compliant with the ASTM International F2952-22 guidelines, we established a rigorous testing framework. In detail, we developed a pump-based test bench and a complementary testing protocol for measuring the permeability of samples under physiologically relevant hydrated conditions and Darcy flow regime. In particular, the proposed PTB relies on a modular permeability chamber (Fig. 1) integrated with a closed-loop hydraulic circuit, which includes a peristaltic pump and pressure sensors coupled with a transducer (Fig. 2). By measuring the pressure drop across the sample and applying Darcy law, the determination of the permeability is achieved. Concurrently, by following the ASTM International F2952-22 guidelines, we developed a specific testing protocol, which allows characterizing the pressure drop directly associated with the tested scaffold minimizing the effects of the uncertainty sources.

To evaluate the operational performance and versatility of the developed PTB, a series of permeability measurements was conducted on different scaffold types using two different test benches (Fig. 3). In detail, 3D-printed PLA scaffolds with regular (PS) and random (RS) structures and commercial scaffolds (CS) for bone TE (Fig. 4) were tested using both the PTB and an alternative test bench (ATB) based on acoustic measurements, implementing a blind randomized testing procedure. Permeability values obtained through both the PTB and the ATB were found to be consistent across all the samples (*E*_*n*_ < 1, Table 2 and Fig. 5), demonstrating the reliability of the proposed test bench. Moreover, regarding the PLA scaffolds, whose dimensional accuracy was preliminarily verified, samples with the same structure (guaranteed by specific and unique G-code files driving the 3D-printer) were characterized by similar permeability values, confirming the proposed PTB’s measurement repeatability (Fig. 5a, b). Diversely, the commercial samples CS1 and CS2 were characterized by different permeability values (Fig. 5c), reflecting the structural differences that arise from their biological origin. Further validation of the PTB in terms of measurement reliability was provided by the agreement between the measured permeability values of the PS scaffolds and their theory-based permeability value (Table 2, Fig. 5a), calculated using the Kozeny-Carman equation. Thanks to the performed tests, it was also possible to confirm that the modular design of the PTB, together with the customized flexible gaskets, guarantees ease of use and it is adaptable to test scaffolds of different geometries and dimensions (height = 1–14 mm, diameter or side = 8–27 mm). The selected pump-based architecture enables the imposition of controlled flow rates, ensuring the establishment of a Darcy flow regime within the samples and making it feasible to test scaffolds over a wide permeability range and under conditions akin to real-world usage scenarios. Moreover, the proposed approach allows overcoming the main drawbacks of the gravity-based set-ups, for which the flow regime is unknown a priori and that are unsuitable for samples characterized by low permeability values^29^. Besides assessing the suitability of the developed permeability test bench, the conducted tests also revealed that certain tested scaffolds were characterized by bone-like permeability values. Interestingly, CS and RS samples showed permeability values consistent with the ones reported in literature for bovine and human trabecular bone^59–62^. Taking into account the intended utilization of the RS scaffolds within perfusion bioreactors for the generation of biomimetic in vitro bone tissue models^63^, these measurements offer additional confirmation of their biomimetic features, which were previously verified through biological testing^64^.

Considering the architecture and components selected for the development of the PTB, some limitations should be acknowledged. First, the peristaltic pump develops a pulsatile flow, which influences the pressure drop measurement. To obtain stable average pressure values, appropriate time periods were tested and defined for the measurement protocol ( "Permeability measurement procedure using the PTB" and Supplementary Fig. S1). Although a syringe pump would have guaranteed stationary flow^33,41^, the choice of a peristaltic pump allowed to build a closed-loop hydraulic circuit for conducting long-term tests, useful for evaluating the possible variation over time of the scaffold permeability due its degradation^22^. Secondly, a wide measurement range characterizes the selected pressure sensors ("Permeability measurements and comparisons"). For this specific study, the pressure values recorded across the tested scaffolds under Darcy flow regime fell in the lower end of the measurement range, limiting measurement accuracy, therefore causing larger uncertainties of permeability measurements obtained with the PTB compared to measurements with the ATB (Table 2). Although for the tested scaffolds the use of pressure sensors with a smaller measurement range would be advisable, the sensitivity of the selected transducers ensured to obtain reliable measurements (Table 2) and the wide measurement range allows maintaining the same architecture for testing different biological materials and scaffolds under different flow regimes. Moreover, since the selected pressure sensors are biocompatible, the PTB architecture could be integrated in perfusion bioreactors^63,65,66^, enabling an indirect evaluation of the structural modification of the cultured constructs by permeability measurements. Regarding the conducted experiments, in this study, we measured scaffold permeability by applying a single flow rate value. Although performing pressure measurements under the application of different flow rates would offer an experimental verification of the development of Darcy flow regime, the overall testing procedure would be highly time consuming. By calculating Reynolds number, the flow regime was verified analytically, allowing the development of a time efficient measurement protocol. In this study, we performed short-term tests and we used demineralize water since the main objective was to assess the performances of the developed PTB. In the future, long-term tests will be performed on degradable scaffolds for testing the influence of their degradation on the permeability values over time.

Finally, considering the intended use of the PTB as a support tool for optimized scaffold manufacturing, it should be noted that, during the scaffold fabrication process, the dimensional accuracy could represent a challenge and affect the permeability evaluation. Indeed, the processing conditions might result in unfaithful manufacturing with respect to the intended scaffold design, dimensions, and performance and, depending on the approach adopted for evaluating the scaffold permeability, this latter could be strongly affected. However, the proposed test bench and measurement procedure, being entirely experimental and based on scaffold geometrical features measured after the manufacturing process, allow the determination of the actual permeability coefficient of the samples, regardless of their dimensional accuracy. In the framework of metrological validation, it is worth mentioning the lack of reference standards for permeability, since nowadays neither certified reference materials, nor reference measurement procedures are available, as defined by the BIPM. Therefore, the "trueness" of the permeability measurements can only be supported from the comparability and compatibility of results, and from the accuracy and precision of the adopted experimental methods, on the basis of interlaboratory comparisons^50^. In literature, methods for permeability measurement were mainly validated by comparison with either theoretical evaluation or with computational fluid dynamics analysis^9,19,32,67,68^. Only Mohee et al.^27^ compared permeability measurements on collagen scaffolds using two distinct experimental set-ups relying on the gravity-based and pump-based methods, respectively. However, the gravity-based method induced the deformation of the samples and different flow rates were adopted, making the results not directly comparable. Moreover, the study was devoted to the measurement of the permeability of a unique type of scaffold. Similarly, other studies performed measurements on a specific scaffold^31^, which in some cases was purposely modified to fit inside the measurement system^19^, or on different scaffolds made of the same materials or using the same fabrication technique^28,33^.

Differing from what has been performed so far, in this study we developed a versatile pump-based permeability test bench and measured the permeability of three types of TE scaffolds, characterized by different structures and compositions and manufactured by using diverse techniques, and we finally compared the results with an alternative experimental method. The compatibility of intrinsic permeability measurements of different scaffolds performed using two different experimental methods based on different test fluids (i.e., demineralized water and air) corroborates the robustness of the proposed approach and constitutes a validation for both test benches and protocols. Moreover, the obtained results were used to confirm the dimensional repeatability of the adopted manufacturing technique, by comparing the permeability values of scaffolds fabricated with the same nominal design parameters. In this context, the proposed approach is suitable for obtaining a reliable and affordable quality assurance procedure for consistent scaffold manufacturing processes, also considering its cost-effectiveness with respect to technologies for microstructural analysis (such as electron microscopy or micro-CT).

With the aim of promoting the dissemination and use of the approach here described and in view of facilitating the cross-laboratory validation and comparability of the scaffold permeability measurements, the design files of the PTB components will be openly provided upon request. Interested researchers are encouraged to request the files, which will be promptly shared to support further exploration, validation, and collaborative developments.

In conclusion, the versatility of the developed PTB, which allows testing porous scaffolds of different types, geometries, and dimensions under different controlled flow regimes, constitutes a significant advantage in view of providing a reliable and repeatable framework for characterizing scaffolds for TE applications, which can span over a wide range of permeability values. Moreover, the application of the measurement protocol described here could be embedded in the TE scaffold design and development process in view of a data-driven refinement of their structural characteristics. In the future, the integration of the proposed approach in perfusion bioreactors could complement the current TE approaches with a real-time non-destructive monitoring of the in vitro model under development.

### Supplementary Information

Supplementary Information.

## Data Availability

The raw data supporting the conclusions of this article will be made available by the corresponding author, without undue reservation, to any qualified researcher.

## References

[1] Vacanti JP, Langer R (1999). Tissue engineering: The design and fabrication of living replacement devices for surgical reconstruction and transplantation. Lancet.

[2] Amini AR, Laurencin CT, Nukavarapu SP (2012). Bone tissue engineering: Recent advances and challenges. Crit. Rev. Biomed. Eng..

[3] Truskey GA, Yuan F, Katz DF (2004). Transport Phenomena in Biological Systems.

[4] Hollister SJ (2005). Porous scaffold design for tissue engineering. Nat. Mater..

[5] O’Brien, F. J. *et al.* The effect of pore size on permeability and cell attachment in collagen scaffolds for tissue engineering. *Technol. Health Care Off. J. Eur. Soc. Eng. Med.* **15**, 3–17 (2007). 17264409

[6] Abbasi N, Hamlet S, Love RM, Nguyen N-T (2020). Porous scaffolds for bone regeneration. J. Sci. Adv. Mater. Dev..

[7] Mattei G, Magliaro C, Pirone A, Ahluwalia A (2018). Bioinspired liver scaffold design criteria. Organogenesis.

[8] Zhao F, Lacroix D, Ito K, van Rietbergen B, Hofmann S (2020). Changes in scaffold porosity during bone tissue engineering in perfusion bioreactors considerably affect cellular mechanical stimulation for mineralization. Bone Rep..

[9] Dias MR, Fernandes PR, Guedes JM, Hollister SJ (2012). Permeability analysis of scaffolds for bone tissue engineering. J. Biomech..

[10] Kelly CN, Miller AT, Hollister SJ, Guldberg RE, Gall K (2018). Design and structure-function characterization of 3D printed synthetic porous biomaterials for tissue engineering. Adv. Healthc. Mater..

[11] Chor MV, Li W (2007). A permeability measurement system for tissue engineering scaffolds. Meas. Sci. Technol..

[12] Massai D (2014). Image-based three-dimensional analysis to characterize the texture of porous scaffolds. BioMed Res. Int..

[13] Li S, de Wijn JR, Li J, Layrolle P, de Groot K (2003). Macroporous biphasic calcium phosphate scaffold with high permeability/porosity ratio. Tissue Eng..

[14] Nield DA, Bejan A (2006). Convection in Porous Media.

[15] Rahbari A, Montazerian H, Davoodi E, Homayoonfar S (2017). Predicting permeability of regular tissue engineering scaffolds: Scaling analysis of pore architecture, scaffold length, and fluid flow rate effects. Comput. Methods Biomech. Biomed. Eng..

[16] Vossenberg P, Higuera GA, van Straten G, van Blitterswijk CA, van Boxtel AJB (2009). Darcian permeability constant as indicator for shear stresses in regular scaffold systems for tissue engineering. Biomech. Model. Mechanobiol..

[17] Agrawal CM, McKinney JS, Lanctot D, Athanasiou KA (2000). Effects of fluid flow on the in vitro degradation kinetics of biodegradable scaffolds for tissue engineering. Biomaterials.

[18] Nasrollahzadeh N, Applegate LA, Pioletti DP (2017). Development of an effective cell seeding technique: Simulation, implementation, and analysis of contributing factors. Tissue Eng. Part C Methods.

[19] Truscello S (2012). Prediction of permeability of regular scaffolds for skeletal tissue engineering: A combined computational and experimental study. Acta Biomater..

[20] Wittkowske C, Reilly GC, Lacroix D, Perrault CM (2016). In vitro bone cell models: Impact of fluid shear stress on bone formation. Front. Bioeng. Biotechnol..

[21] Fan J, Jia X, Huang Y, Fu BM, Fan Y (2015). Greater scaffold permeability promotes growth of osteoblastic cells in a perfused bioreactor. J. Tissue Eng. Regen. Med..

[22] Jeong, C. G. & Hollister, S. J. Mechanical, permeability, and degradation properties of 3D designed poly(1,8 octanediol-co-citrate) scaffolds for soft tissue engineering. *J. Biomed. Mater. Res. B Appl. Biomater.* **93B**, 141–149 (2010). 10.1002/jbm.b.31568 PMC4369673 20091910

[23] Pennella F (2013). A survey of methods for the evaluation of tissue engineering scaffold permeability. Ann. Biomed. Eng..

[24] Innocentini MDM (2010). Permeability of porous gelcast scaffolds for bone tissue engineering. J. Porous Mater..

[25] Wang Y, Tomlins PE, Coombes AGA, Rides M (2010). On the determination of Darcy permeability coefficients for a microporous tissue scaffold. Tissue Eng. Part C Methods.

[26] Offeddu GS, Ashworth JC, Cameron RE, Oyen ML (2016). Structural determinants of hydration, mechanics and fluid flow in freeze-dried collagen scaffolds. Acta Biomater..

[27] Mohee L, Offeddu GS, Husmann A, Oyen ML, Cameron RE (2019). Investigation of the intrinsic permeability of ice-templated collagen scaffolds as a function of their structural and mechanical properties. Acta Biomater..

[28] Lipowiecki M (2014). Permeability of rapid prototyped artificial bone scaffold structures. J. Biomed. Mater. Res. A.

[29] Nasrollahzadeh N, Pioletti DP (2016). Experimental method to characterize the strain dependent permeability of tissue engineering scaffolds. J. Biomech..

[30] Sanz-Herrera, J. A. *et al.* Mechanical and flow characterization of Sponceram^®^ carriers: Evaluation by homogenization theory and experimental validation. *J. Biomed. Mater. Res. B Appl. Biomater.* **87B**, 42–48 (2008). 10.1002/jbm.b.31065 18395821

[31] Ochoa I (2009). Permeability evaluation of 45S5 Bioglass^®^-based scaffolds for bone tissue engineering. J. Biomech..

[32] Podichetty JT, Bhaskar PR, Khalf A, Madihally SV (2014). Modeling pressure drop using generalized scaffold characteristics in an axial-flow bioreactor for soft tissue regeneration. Ann. Biomed. Eng..

[33] Santos J, Pires T, Gouveia BP, Castro APG, Fernandes PR (2020). On the permeability of TPMS scaffolds. J. Mech. Behav. Biomed. Mater..

[34] ASTM International F2952–22. *Guide for Determining the Mean Darcy Permeability Coefficient for a Porous Tissue Scaffold*. 10.1520/F2952-22 (2022).

[35] Schiavi A, Guglielmone C, Pennella F, Morbiducci U (2012). Acoustic method for permeability measurement of tissue-engineering scaffold. Meas. Sci. Technol..

[36] Fiume E (2021). Comprehensive assessment of bioactive glass and glass-ceramic scaffold permeability: Experimental measurements by pressure wave drop, modelling and computed tomography-based analysis. Acta Biomater..

[37] Fernandez M, Vink J, Yoshida K, Wapner R, Myers KM (2013). Direct measurement of the permeability of human cervical tissue. J. Biomech. Eng..

[38] Fujie H, Imade K (2015). Effects of low tangential permeability in the superficial layer on the frictional property of articular cartilage. Biosurf. Biotribol..

[39] Benalla M, Palacio-Mancheno PE, Fritton SP, Cardoso L, Cowin SC (2014). Dynamic permeability of the lacunar–canalicular system in human cortical bone. Biomech. Model. Mechanobiol..

[40] Grimm MJ, Williams JL (1997). Measurements of permeability in human calcaneal trabecular bone. J. Biomech..

[41] Kleinhans KL, Jackson AR (2018). Hydraulic permeability of meniscus fibrocartilage measured via direct permeation: Effects of tissue anisotropy, water volume content, and compressive strain. J. Biomech..

[42] Pecci R, Baiguera S, Ioppolo P, Bedini R, Del Gaudio C (2020). 3D printed scaffolds with random microarchitecture for bone tissue engineering applications: Manufacturing and characterization. J. Mech. Behav. Biomed. Mater..

[43] Zenobi E (2023). Tailoring the microarchitectures of 3D printed bone-like scaffolds for tissue engineering applications. Bioengineering.

[44] Perale G (2019). Biomimetic biomolecules in next generation xeno-hybrid bone graft material show enhanced in vitro bone cells response. J. Clin. Med..

[45] Bear J (1988). Dynamics of Fluids in Porous Media.

[46] Fand RM, Kim BYK, Lam ACC, Phan RT (1987). Resistance to the flow of fluids through simple and complex porous media whose matrices are composed of randomly packed spheres. J. Fluids Eng..

[47] Dybbs, A. & Edwards, R. V. A new look at porous media fluid mechanics—Darcy to turbulent. In *Fundamentals of Transport Phenomena in Porous Media* (eds. Bear, J. & Corapcioglu, M. Y.). 199–256 10.1007/978-94-009-6175-3_4 (Springer Netherlands, 1984).

[48] JCGM 100:2008 (GUM 1995 with minor corrections). *Evaluation of Measurement Data—Guide to the Expression of Uncertainty in Measurement* (2008).

[49] ISO/IEC 17043:2023. *Conformity Assessment. General Requirements for the Competence of Proficiency Testing Providers* (2023).

[50] JCGM 200:2012. *International Vocabulary of Metrology—Basic and General Concepts and Associated Terms* (*VIM*) (2012).

[51] Kozeny J (1927). Uber Kapillare Leitung der Wasser in Boden. R. Acad. Sci. Vienna Proc. Cl..

[52] Carman PC (1937). Fluid flow through granular beds. Chem. Eng. Res. Des..

[53] Mauret E, Renaud M (1997). Transport phenomena in multi-particle systems—I. Limits of applicability of capillary model in high voidage beds-application to fixed beds of fibers and fluidized beds of spheres. Chem. Eng. Sci..

[54] Ghanbarian B, Hunt AG, Ewing RP, Sahimi M (2013). Tortuosity in porous media: A critical review. Soil Sci. Soc. Am. J..

[55] Comiti J, Renaud M (1989). A new model for determining mean structure parameters of fixed beds from pressure drop measurements: Application to beds packed with parallelepipedal particles. Chem. Eng. Sci..

[56] Hussaini SR, Dvorkin J (2021). Specific surface area versus porosity from digital images. J. Pet. Sci. Eng..

[57] Nakayama A, Kuwahara F, Sano Y (2007). Concept of equivalent diameter for heat and fluid flow in porous media. AIChE J..

[58] Lam RC, Kardos JL (1991). The permeability and compressibility of aligned and cross-plied carbon fiber beds during processing of composites. Polym. Eng. Sci..

[59] Kohles SS (2001). Direct perfusion measurements of cancellous bone anisotropic permeability. J. Biomech..

[60] Prakoso AT (2023). The effect of tortuosity on permeability of porous scaffold. Biomedicines.

[61] Lim T-H, Hong JH (2000). Poroelastic properties of bovine vertebral trabecular bone. J. Orthop. Res..

[62] Nauman EA, Fong KE, Keaveny TM (1999). Dependence of intertrabecular permeability on flow direction and anatomic site. Ann. Biomed. Eng..

[63] Gabetti S (2022). An automated 3D-printed perfusion bioreactor combinable with pulsed electromagnetic field stimulators for bone tissue investigations. Sci. Rep..

[64] Ledda M (2022). Biological response to bioinspired microporous 3D-printed scaffolds for bone tissue engineering. Int. J. Mol. Sci..

[65] Yamada S, Yassin MA, Schwarz T, Mustafa K, Hansmann J (2022). Optimization and validation of a custom-designed perfusion bioreactor for bone tissue engineering: Flow assessment and optimal culture environmental conditions. Front. Bioeng. Biotechnol..

[66] Born G (2022). Mini- and macro-scale direct perfusion bioreactors with optimized flow for engineering 3D tissues. Biotechnol. J..

[67] Pires T (2021). Numerical–experimental analysis of the permeability–porosity relationship in triply periodic minimal surfaces scaffolds. J. Biomech..

[68] d’Adamo, A. *et al.* Experimental measurements and CFD modelling of hydroxyapatite scaffolds in perfusion bioreactors for bone regeneration. *Regen. Biomater.* **10**, rbad002 (2023). 10.1093/rb/rbad002 PMC9893872 36751469

