# Supplementary material for: Adaptable test bench for ASTM-compliant permeability measurement of porous scaffolds for tissue engineering
Source: Sci Rep. 2024 Jan 19;14:1722. doi: 10.1038/s41598-024-52159-4 (PMC10799031; doi:10.1038/s41598-024-52159-4)
Supplement: Supplementary file 1 — Supplementary Information. [file 41598_2024_52159_MOESM1_ESM.pdf]

Supplementary materials

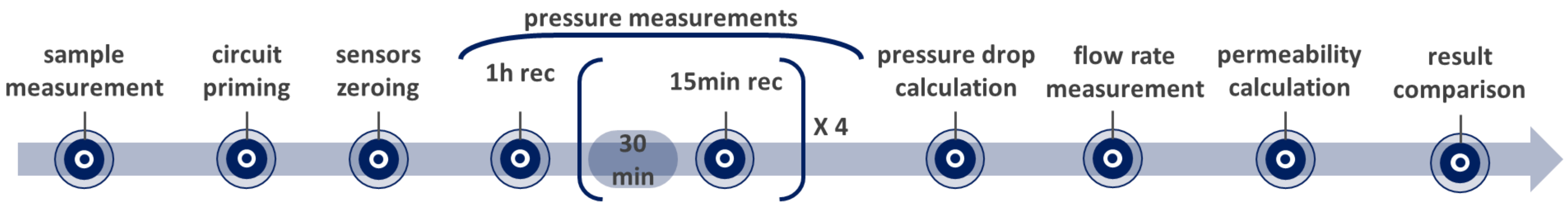

Supplementary Figure S1. Detailed timeline of the permeability measurement procedure using the PTB.

Acoustic test bench architecture and test procedure

Permeability measurements were conducted on all the samples by means of a calibrated acoustic permeameter developed for measurements in dry-air conditions (ATB) for medium/high permeable materials (10^{-14} - 10^{-8} m^2), as described in detail in Schiavi et al[1]. The ATB provides a rapid permeability measurement, based on the accurate quantification of the acoustic pressure wave drop of an alternating airflow through the samples, generated by an oscillating piston in a cavity (Supplementary Fig. S2a), by using a single low-frequency pressure field microphone (Supplementary Fig. S2b).

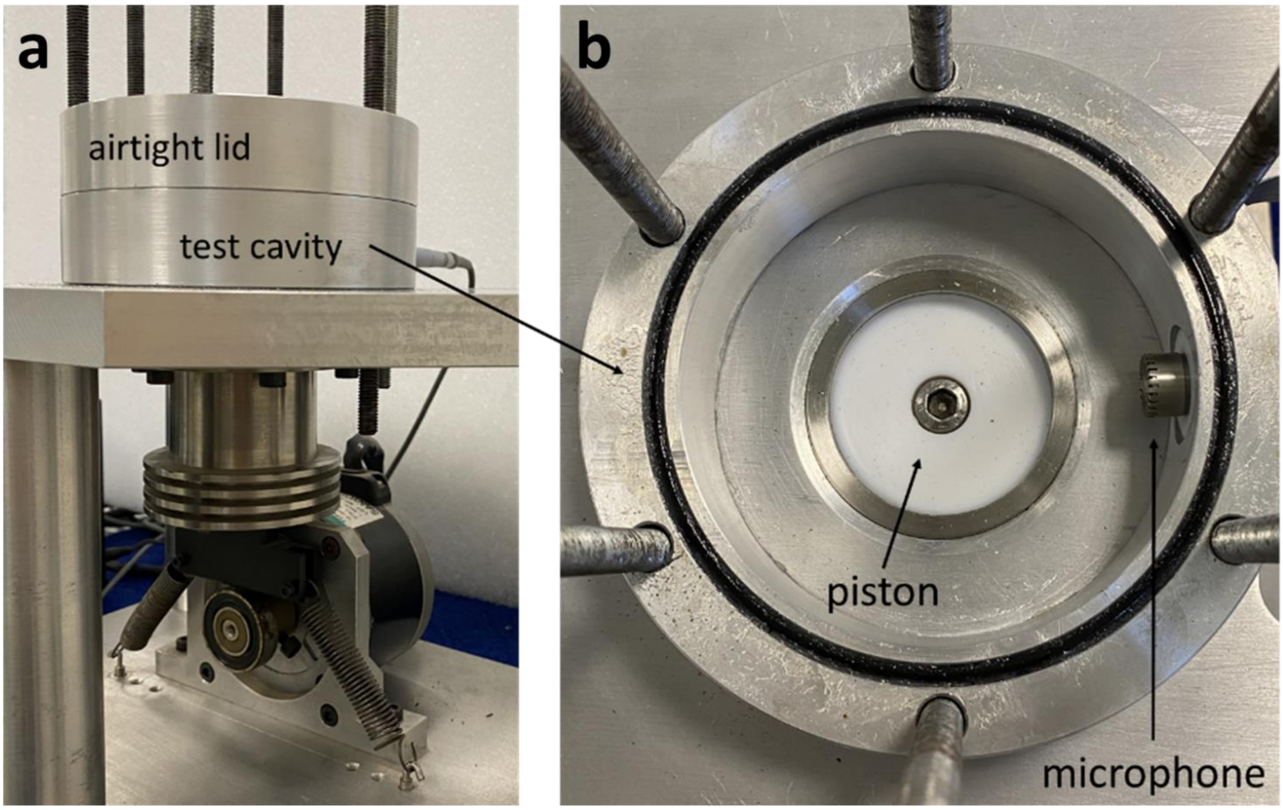

Supplementary Figure S2. (a) ATB setup. (b) detail of the ATB test cavity with the piston and the microphone.

As a first step, the sensitivity of the microphone (B&K ½" microphone, type 4193) is determined with a calibration method, as schematically shown in Supplementary Figure 3a. The test cavity is closed by a full airtight lid and by means of the piston (driven by a crank-connecting rod system, managed by a stepper motor with accuracy of $1.6 \cdot 10^{-3}$ $rad \cdot s^{-1}$ /step), very slow pulsations (w ~ 1 $rad \cdot s^{-1}$) of the air volume V_0 in the test cavity are generated, inducing pressure oscillations with wave amplitude of:

$$p_{\mathrm{rms}} = \frac{\gamma p_0 \partial V}{V_0 \sqrt{2}} \quad (1)$$

where p_0 is the atmospheric pressure (with heat capacity ratio $\gamma = 1.4$), and ∂V is the volume variation induced by the motion of the piston. The occurring pressure waves act on the microphone membrane generating a proportional voltage variation, which is amplified (G.R.A.S. Power Module, type 12AK), acquired by a DAQ (USB4431, National Instruments) and analyzed in terms of angular frequency and amplitude. The ratio between the measured output voltage and the pressure waves amplitude provides the microphone sensitivity, expressed in mV/Pa_{cal}.

Then, the full airtight lid is removed and replaced by an airtight lid with a passing hole closed by the porous/permeable sample to be investigated. To avoid losses, all junctions are sealed with O-

rings held under pressure by passing screws. In addition, a thin layer of petroleum jelly closes any possible further leakage in the seals. The samples are located in a specific holder in Teflon, sealed with a Teflon tape; a further plasticine seal was placed between the sample and the holder, to avoid possible leakage at the edge.
The pulsations of the air volume V_0 through the samples, generate an alternate flow rate

$$q_{v,\mathrm{rms}} = \frac{\omega \partial V}{\sqrt{2}} \quad (2)$$

low enough to keep interstitial Reynolds number close to unit. The amplitude of the pressure wave, measured by the microphone is reduced with respect to the calibration conditions, since a certain quantity of air is slowly pumped in and out through the porous sample, as schematically shown in Supplementary Figure S3b.

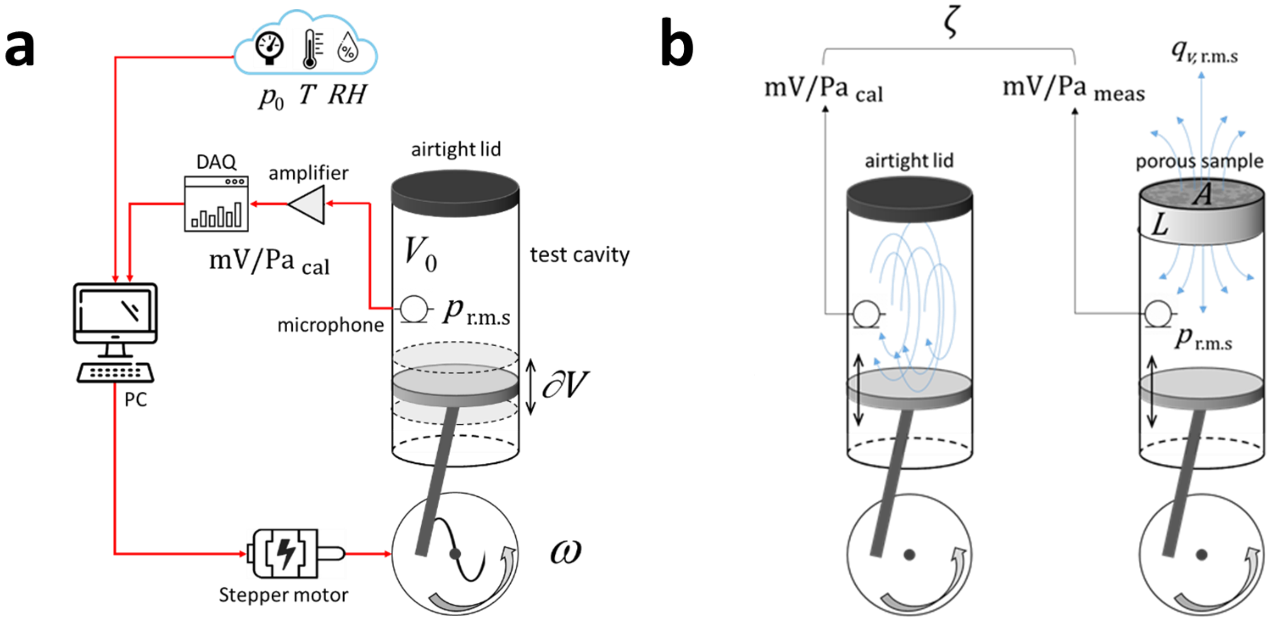

Supplementary Figure S3. (a) calibration of the microphone within the test cavity of the ATB. (b) conceptualization of the intrinsic permeability measurement method by using the ATB.

The pressure wave drop is then determined by the ratio ζ between the amplitude of the pressure wave measured in the hermetically closed air volume (i.e., the microphone sensitivity), and the amplitude of the pressure wave measured in the same volume of air enclosed by the porous/permeable sample (mV/Pa$_{\mathrm{meas}}$). Thus, intrinsic permeability k_D is experimentally determined from the ratio between the r.m.s. volumetric airflow rate, and the r.m.s. dynamic pressure, according to Darcy's law for oscillating flows, as follows:

$$k_D = \mu \frac{Q}{\Delta P} \cdot \frac{L}{A} = \mu \frac{q_{v,\mathrm{rms}}}{p_{\mathrm{rms}}} \cdot \frac{L}{A} \cdot \zeta = \mu \frac{\omega \partial V (\sqrt{2})^{-1}}{\gamma p_0 \partial V (V_0 \sqrt{2})^{-1}} \cdot \frac{L}{A} \cdot \zeta = \mu \frac{\omega V_0}{\gamma p_0} \cdot \frac{L}{A} \cdot \zeta \qquad (3)$$

where μ is the dynamic viscosity μ of the air.
The measurements are carried out at a constant airflow pulsation $w = (0.926 \pm 0.003)$ rad/s. The volume of air confined in the test cavity is $V_0 = (2.498 \pm 0.009) \cdot 10^{-4}$ m^3, and the volumetric airflow results $q_{v,\mathrm{rms}} = 1.07 \cdot 10^{-6}$ m^3/s, since $\partial V = 1.64 \cdot 10^{-6}$ m^3 (with a peak-to-peak stroke displacement of $1.71 \cdot 10^{-3}$ m, and a piston surface area of $1.92 \cdot 10^{-3}$ m^2). The atmospheric static pressure is measured by a barometer (Druck DPI 280), with a fractional accuracy of $1 \cdot 10^{-4}$, in the range 800-1100 mbar.
For each tested sample, by accurately measuring the relative amplitudes of the alternating pressure waves, the geometrical dimensions of the sample under investigation, and the atmospheric static pressure during the measurement, the permeability can be evaluated.

Table S1 reports the measured and calculated parameters for permeability measurement of a specific sample (scaffold CS1), along with relevant standard uncertainties. The same parameters were also evaluated for the permeability values of all measurements, whose results in terms of permeability and expanded uncertainty are reported in Table 1 of the main manuscript.

Table S1. Measured and calculated parameters for all scaffold CS1 by using the ATB

		Parameter	CS1
Measurement	*sample*	$L \pm u^2$ (m)	$3.47 \cdot 10^{-3} \pm 6.3 \cdot 10^{-10}$
		$A \pm u^2$ (m^2)	$8.08 \cdot 10^{-5} \pm 7.8 \cdot 10^{-12}$
	ATB	$\mu \pm u^2$ (Pa·s)	$1.81 \cdot 10^{-5} \pm 3.3 \cdot 10^{-17}$
		$\omega \pm u^2$ (rad/s)	$9.26 \cdot 10^{-1} \pm 1.3 \cdot 10^{-5}$
		$V_0 \pm u^2$ (m^3)	$2.49 \cdot 10^{-4} \pm 2.1 \cdot 10^{-13}$
		$p_0 \pm u^2$ (Pa)	$9.78 \cdot 10^{4} \pm 33.3$
		$Volt_{cal} \pm u^2$	$1.34 \pm 1.1 \cdot 10^{-7}$
		$Volt_{test} \pm u^2$	$6.68 \cdot 10^{-2} \pm 2.9 \cdot 10^{-6}$
Calculation	*ATB*	$k \pm U_k$ (m^2)	$(2.63 \pm 0.226) \cdot 10^{-11}$

Flexible gasket manufacturing

To guarantee the watertightness of the sample clamping within the PC, the sample is inserted in a tailored flexible gasket, appositely manufactured. In detail, depending on the sample geometry and size, the gasket is designed with a tailored central channel for housing the sample. Moreover, it presents two rectangular grooves on opposite sides to facilitate the gasket extraction from the PC with the use of pliers.
For the manufacturing of flexible gaskets suitable for housing samples of different geometries and sizes, and thus with different internal channels, a modular mould with interchangeable spacers were designed (Solidworks, Dassault Systemes, France) and fabricated by fused deposition modelling (3D printer uPrint SE, Stratasys, USA). The mould is composed of interlocking top and bottom parts and of an interchangeable spacer (Figure S4a). The inner surface of the top part of the mould presents ridges to produce the lateral grooves of the gaskets. By using the interchangeable spacers, it is possible to obtain different tailored gaskets for housing samples of different geometries and sizes, guaranteeing the versatility of the PTB. For this specific study, cylindrical scaffold samples were tested and a set of 4 spacers with different external diameters (9.5, 9.7, 9.8, 10 mm) were manufactured. After assembling the mould (Figure S4b), the tailored gaskets were manufactured by pouring a two-component liquid silicone rubber (R Pro Tech 33, Reschimica, Italy) in the mould and allowing it to solidify at room temperature for three days (Figure S4c).

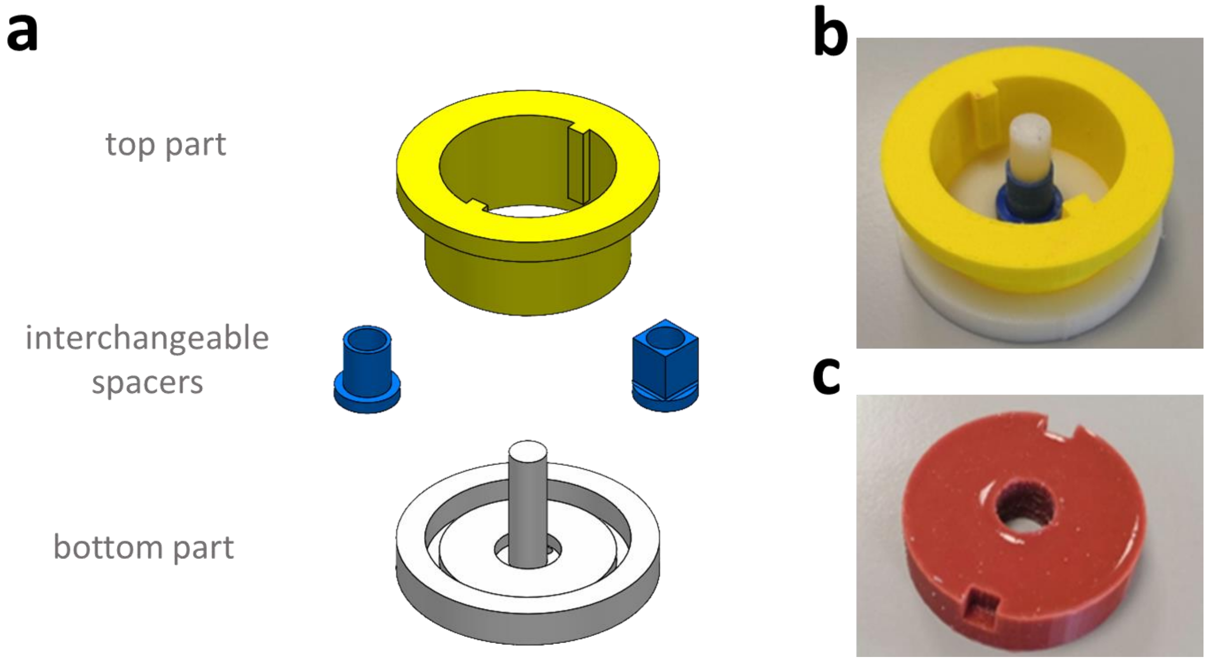

Supplementary Figure S4. (a) CAD view of the modular mould components. (b) Picture of the assembled mould components: 3D-printed top part, bottom part, and interchangeable spacer. (c) Manufactured flexible gasket.

Measurement of PS scaffold porosity

The porosity of the PS scaffolds was measured performing a gravimetric analysis. The scaffolds were weighted with an electronic balance (Soptop FA1204) to evaluate their mass (m_i).

Knowing the nominal overall volume of the scaffolds ($V = 300$ mm^3), the density for each scaffold model was calculated as:

$$\rho_i = m_i \cdot V \qquad (4)$$

Then, the mean percentage porosity (φ) was calculated, knowing the PLA density ($\varrho_{PLA} = 1240$ kg/m^3), by means of the following equation:

$$\varphi = \frac{1 - \rho_i}{\rho_{PLA}} \cdot 100 \qquad (5)$$

Determination of ***a*** coefficient for analytical evaluation of permeability

The logarithmic model described in Section 2.4.1 allows to compute scaffold tortuosity from the equation:

$$\tau = 1 - a \cdot ln(\varphi) \qquad (6)$$

where a is a coefficient depending on the internal structure of the porous material and φ is the material porosity. The a coefficient was computed using the equation proposed by Comiti and Renaud[2] for porous medium composed by parallelepipedal particles:

$$a = 0.58 \cdot e^{0.18 \cdot t/w} \qquad (7)$$

where t is the thickness of the particle and w is the width of the particle.
Considering that the particles of the PS scaffold have a square cross section, therefore $t = w$, the obtained value for the coefficient a was 0.694.

1. Schiavi, A., Guglielmone, C., Pennella, F. & Morbiducci, U. Acoustic method for permeability measurement of tissue-engineering scaffold. *Meas. Sci. Technol.* 23, 105702 (2012).
2. Comiti, J. & Renaud, M. A new model for determining mean structure parameters of fixed beds from pressure drop measurements: application to beds packed with parallelepipedal particles. *Chem. Eng. Sci.* 44, 1539–1545 (1989).
